# Relation between lipid profile, blood pressure and retinopathy in diabetic patients in King Abdulaziz University hospital: a retrospective record review study

**DOI:** 10.1186/s40942-022-00366-4

**Published:** 2022-03-09

**Authors:** Khadijah Alattas, Dania W. Alsulami, Rahaf H. Alem, Felwa S. Alotaibi, Bayan A. Alghamdi, Layan S. Baeesa

**Affiliations:** 1 Department of Ophthalmology, College of Medicine, King Abdulaziz University Hospital, Jedddah, Saudi Arabia; 2 Faculty of Medicine, King Abdulaziz University, Jeddah, Saudi Arabia

**Keywords:** Diabetic retinopathy, Retinopathy, Lipid profile, DR, Lipid effect

## Abstract

**Background:**

Diabetic retinopathy (DR) is a major cause of blindness worldwide, threatening the vision of approximately 10% of patients with diabetes. Many studies have demonstrated that intensive control of the risk factors for DR is essential to reduce the onset and progression of DR. Currently, the relationship between lipid profile and DR is still unclear, especially in Saudi Arabia. We aimed to assess the correlation between both the development and severity of DR with lipid profile and blood pressure among the diabetic patients at the King Abdul-Aziz University hospital in Jeddah, Saudi Arabia.

**Methods:**

This was a retrospective record review study of 298 diabetic patients diagnosed with DR. Retinal findings were correlated to serum lipids levels using univariate, bivariate, and multivariate analysis.

**Results:**

This study included 298 participants with DR. Triglyceride levels, systolic blood pressure, low-density lipoprotein cholesterol levels, and presence of macular edema were significantly associated with DR progression (P = 0.012, P = 0.001, P = 0.002). Other parameters, including total cholesterol, high-density lipoprotein cholesterol, HbA1C, body mass index, age, were not significantly associated with DR.

**Conclusion:**

Elevation in serum triglyceride levels and systolic blood pressure showed a statically significant association with diabetic retinopathy. Controlling these factors may help preventing progression and occurrence of diabetic retinopathy among diabetic patients.

## Introduction

Diabetic retinopathy (DR) is a microvascular complication of diabetes mellitus (DM) that is an important cause of visual impairment in adults, threatening the vision of approximately 10% of patients with diabetes [1, 2]. Therefore, DR is a growing concern worldwide. As DM complications and morbidity increase, the DR prevalence is predicted to reach 5.4% by 2025 [3]. Identifying and controlling the risk factors for DR is essential to reduce the onset and progression of DR [4]. Several risk factors for DR have been identified, including the duration of DM, glucose level, and blood pressure [5]. Moreover, it has been suggested that patients’ lipid status also affects the onset and pathogenesis of DR [6]. Diabetic retinopathy can be categorized into non-proliferative diabetic retinopathy (NPDR) and proliferative diabetic retinopathy (PDR) based on its stage and severity [7, 8].

Previous studies have reported conflicting results regarding the effect of serum lipids on the onset and progression of DR [9]. Dyslipidemia associated with diabetes is characterized by high serum levels of total cholesterol (TC), triglycerides (TG), low-density lipoprotein cholesterol (LDL-C), and high-density lipoprotein cholesterol (HDL-C), and these are proposed to represent possible markers for DR progression and the occurrence of diabetic macular edema (DME) [9].

Several studies have been conducted to identify factors associated with DR progression. A previous study of 140 patients with type 2 diabetes has conducted to determine the correlation between the severity of DR, and serum lipid and other modifiable risk factors found that high cholesterol level, blood pressure, renal function, and urine albumin excretion are significantly associated with the progression of DR, while there was no significant association between HbA1c and DR [10]. In addition, the Chennai Urban Rural Epidemiological Study by Rema et al. which included 1763 Indian type 2 diabetic subjects suggested that serum triglycerides are associated with the risk of DR, while LDL-C was associated with DME [11]. Regarding other risk factors for DR, Antonetti et al. described the effect of arteriolar dysfunction and hemodynamic alterations on late-stage microvascular disease and vision loss [12].

In contrast, a meta-analysis conducted in 2017 included seven studies that did not find apparent differences in TG, TC, and HDL-C levels between patients with and without DR [3]. Further, a retrospective study that included 191 Japanese participants found no significant correlation between serum lipid levels with the severity of DR or existence of macular edema despite the significant correlation between the blood glucose, HbA1c, and total cholesterol [13].

Although several studies have been conducted on this topic, the relationship between lipid profile, blood pressure, and DR is still unclear, especially in Saudi Arabia. In particular, there are limited studies which have focused on the role of lipids as a risk factor for DR. To this end, our study aimed to assess the correlation between both lipid profile and blood pressure and the development and severity of DR among diabetic patients at the King Abdul-Aziz University Hospital in Jeddah, Saudi Arabia in 2021.

## Methodology

This retrospective record review study was conducted in the Department of Ophthalmology at the King Abdul-Aziz University Hospital in Jeddah, Saudi Arabia, in June 2021. The study was approved by the Research Ethics Committee of King Abdulaziz University (KAU) (538-20). The medical records of 289 diabetic patients with retinopathy were reviewed. The severity of DR was classified into mild non-proliferative DR (NPDR), moderate NPDR, severe NPDR, and PDR, according to the ETDRS grading scale [14]. Patients aged 18 years and above with known type 1 or type 2 diabetes mellitus and diagnosed with DR were included. Patients with a recent eye infection, who had undergone any ocular surgery within the previous 6 months, or those who were pregnant were excluded. Demographic data, such as age, gender, and nationality were collected from the medical records. Moreover, we also collected clinical data, such as systolic blood pressure; diastolic blood pressure; Hba1C; and lipid profile, including TC, TG, low-density lipoprotein (LDL), high-density lipoprotein (HDL), and creatinine levels. Triglycerides and cholesterol classification were according to The National Cholesterol Education Program Adult Treatment Panel III guidelines [15]. Body mass index (BMI) was calculated for each patient using the BMI formula (weight in kg divided by height in meters squared). The primary outcome was the relationship between lipid profile, blood pressure, and DR. eGFR calculated using the 2009 CKD-EPI creatinine equation.

Data were registered using online Google Forms and entered in Microsoft Excel 2016, and statistical analysis was performed using Statistical Package for the Social Science (SPSS) version 21. Means and standard deviations were calculated to describe continuous variables, whereas numbers and percentages were used for categorical variables. Student’s t-test and chi-square test were used to evaluate the differences between categorical variables. Statistical significance was set at p < 0.05. Quantitative data were presented as mean and standard deviation (mean ± SD), where one-way ANOVA and Kruskal Wallis tests were applied according to data normality.

Multiple logistic regression analysis was performed with DR stage as the dependent variable.

## Results

The study included 165 women (57.1%) and 124 men (42.9%). Ninety-six (33.2%) patients had mild NPDR, 103 (35.6%) had moderate NPDR, 29 (10%) had severe NPDR, and 61 (21.1%) had PDR.

Triglyceride levels were significantly associated with the severity of DR; patients with PDR showed significantly higher TG levels than NPDR patients (P = 0.012). Patients with advanced DR stage showed higher LDL levels (Table 1). There was no significant relationship between the progression of DR and cholesterol, DBP, and HBa1C (P = 0.231, P = 0.256, P = 0.197, respectively) (Table 1).

**Table 1:** Clinical characteristic of different stages of diabetic retionpathy

	**Mild DR** **N = 96**	**Moderate DR** **N = 103**	**Severe DR** **N = 29**	**PDR** **N = 61**	**P value**
Age	61.25 ± 13.56	62.52 ± 11.34	61.72 ± 9.03	57.92 ± 12.49	0.132*
BMI	30.26** ± **6.65	31.89 ± 5.94	31.71 ± 5.30	30.88 ± 6.18	0.285*
cholesterol	4.67 ± 1.01	4.85 ± 1.12	5.43 ± 1.41	13.1 ± 60.2	0.231*
triglycerides	1.61 ± 0.78	1.88 ± 1.09	1.94 ± 1.11	2.23 ± 1.58	0.012*
LDL	2.97 ± 1.02	3.06 ± 0.93	3.61 ± 1.21	3.45 ± 1.08	0.005*
HDL	1.28 ± 0.40	1.24 ± 0.51	1.15 ± 0.28	1.29 ± 0.85	0.771*
creatinine	87.68 ± 51.16	83.19 ± 28.76	111.41 ± 149.03	117.83 ± 89.69	0.009*
SBP	139.46 ± 16.58	142.66 ± 17.81	151.03 ± 19.66	149.39 ± 18.30	0.001*
DBP	73.03 ± 9.97	73.36 ± 10.82	76.98 ± 10.78	75.17 ± 11.98	0.256*
HBA1C	8.84 ± 1.62	9.09 ± 1.89	9.25 ± 0.187	9.51 ± 2.38	0.197*

*One-way ANOVA

In our analysis, we found that as SBP increased, the severity of DR also increased (P = 0.001) (Table 1). There was no association between DR stage and creatinine level (P = 0.165). Table 2 shows the results of multinomial logistic regression analysis to assess the independent predictors of DR stage. High TG, high TG category, low mean GFR, and a high mean creatinine level were risk factors for advanced DR stage (p ≤ 0.05).

**Table 2:** Multinominal logistic regression analysis the independent predictors (risk factors) of advanced DR staging

Variable	Likelihood ratio	Chi-square	df	p-value
Age	461.8	6.54	3	0.088
Gender	464.1	8.84	3	0.031
Nationality	458.28	3.03	3	0.388
Macular edema	462.61	7.35	3	0.061
SBPM	456.81	1.55	3	0.669
DBPM	457.58	2.32	3	0.508
Cholesterol	458.23	2.97	3	0.395
Cholesterol categories	458.85	3.58	3	0.309
Triglycerides	465.02	9.76	3	0.021
Triglyceride categories	464.62	9.36	3	0.025
GFR	473.01	17.75	3	< 0.001
Creatinine	465.01	7.95	3	0.021
HbA1c	455.67	0.41	3	0.938
BMI	455.8	0.54	3	0.909
BMI categories	457.03	1.77	3	0.62

Other lipid marker analyses are presented in Table 3. Patients with severe NPDR had significantly higher mean LDL values than those with other DR stages (p = 0.005). However, a non-significant relationship was found between the DR stage and HDL (P = 0.534).

**Table 3:** Relationship between DR stage and HDL and LDL

Variable	Mild NPDR Mean ± SD	Moderate NPDR Mean ± SD	sever NPDR Mean ± SD	PDR Mean ± SD	Test	p-value
HDL	1.27 ± 0.39	1.24 ± 0.51	1.15 ± 0.28	1.29 ± 0.84	3*	0.534
LDL	2.97 ± 1.02	3.05 ± 0.93	3.6 ± 1.21	3.44 ± 1.07	4.44**	0.005

N.B.: * Kruskal Wallis test **One Way ANOVA test

## Discussion

The reported prevalence of DR in Saudi Arabia in 2019 was 33.7% [16]. Diabetes and its complications pose a significant personal and public health burden; therefore, identifying and modifying the risk factors for diabetes is an important clinical goal. Numerous studies have found a correlation between lipid fractions and macrovascular complications of diabetes (e.g., coronary artery disease) [17]. However, very few studies have focused on the association between serum lipids and microvascular complications such as DR. Our study aimed to assess the association between DR severity, lipid profile, and blood pressure among diabetic patients at the King Abdul-Aziz University Hospital in Jeddah.

Our analysis showed a significant relationship between TG levels and DR severity, which is comparable to findings reported in India which showed a strong positive relationship between TG and DR stage [10]. The role of serum lipids in the development and progression of DR has been evaluated worldwide; hyperlipidemia causes endothelial dysfunction due to reduced bioavailability of nitric oxide and breakdown of the blood retinal barrier, which leads to exudation of serum lipids and lipoproteins, resulting in DR changes [9].

Other studies have also investigated the relationship between DR and other lipid parameters. Unlike previous studies which demonstrated an association between cholesterol and DR [10], we found no significant association between serum cholesterol and HDL with DR stage. Our observations that there was no association between DR stage and cholesterol and HDL were consistent with those of previous reports from Turkey and India [13, 18] and those of the WESDR study [19]. This observation is consistent with the mechanism of DR progression which is thought to be related to intraretinal lipid transportation, rather than serum lipid levels per se [13].

In our study, systolic blood pressure was associated with the presence and severity of DR. This result was consistent with the results of previous studies [5, 10, 20]. Hypertensive patients had a more than two-fold risk of DR compared to diabetic patients with controlled blood pressure. This may be because the endothelium of the retinal capillaries is injured in DR, and hypertension promotes endothelial disturbance [5].

This study has some limitations worth noting. First, this was single-center study and therefore further studies are warranted to confirm whether the results are translatable to other healthcare settings. Second, the difference in time of laboratory readings could influence the results. Therefore, further prospective studies are warranted to validate our results. Also, the small sample size due to single-center data consider an important limitation to mention and the number of included participants could be greater. Nevertheless, the results described herein are valuable as this is one of the first studies conducted to assess the correlation between both causation and severity of DR with lipid profile levels and blood pressure in Saudi Arabia.

## Conclusion

In conclusion, our findings have clear clinical implications. The association of serum lipids, particularly triglyceride levels, with DR severity and progression suggests that hyperlipidemia may have an impact on DR, despite the lack of association with other lipid parameters such as cholesterol and HDL. We also found that systolic blood pressure was associated with DR progression. These observations help to clarify some of the discrepancies of previous studies and suggest that lipid-targeting therapies may be more effective in slowing the progression of DR than in preventing the development of DR per se. Taken together, these results demonstrate the importance of measuring lipid levels and blood pressure in patients with diabetes to initiate appropriate treatments and prevent the onset and progression of diabetic retinopathy and DME.

## Data Availability

Raw data were generated at King Abdul-Aziz University Hospital.Derived data supporting the findings of this study are available from the corresponding author D. W. A. on request.
